# Nutritional status in idiopathic scoliosis

**DOI:** 10.1186/1748-7161-7-S1-O22

**Published:** 2012-01-27

**Authors:** J Durmala, M Sosnowska, M Sosnowski

**Affiliations:** 1 School of Health Care Katowice, Poland; 2 School of Medicine, Medical University of Silesia, Katowice, Poland

## Background

A relatively high proportion of lean body habitus is an acknowledged feature of idiopathic scoliosis [1]. We aimed at evaluation of nutritional status in schoolchildren with idiopathic scoliosis managed in a single center of rehabilitation. Data from a homogeneous population regarding current national BMI reference is presented.

## Materials and methods

303 children were included. There were 260 girls and 43 boys, aged 14.2±0.2 and 14.1±0.4 years, resp. In each girl or boy, the body height and mass was measured by using the standardized protocol. Individual BMI (kg/m2) was classified according to the established normal age- and gender-related range (Z-score) of limit for Polish children. The calculations were performed on basis of actually measured height (BMI), as well as after height correction (BMIcor). Data were compared with the BMI distribution in healthy children.

## Results

The BMI values lower than 2SD Z-score and 1SD Z-score were found in 3.3% and 17.8%. The BMI values higher than 2SD and 1SD were found in 0.3% and 7.9%, resp. After correction for height, the proportions for low BMIcor were 5.0% and 24.0%. There was none case of obesity after height-correction, and the proportion of overweight children reached 6.0%. Compared to BMI in normal population, the frequency of low BMI or BMIcor in IS was found 3.05- or 4.2-times higher, resp. On contrary, the frequency of high BMI or BMIcor was 2.0 or 2.7-times lower, resp.

## Conclusions

Almost one-third of children with IS are underweight, while obesity is a sporadic feature. Reasons of low nutritional status should be explained in each case.
